# Elements of musical and dance sophistication predict musical groove perception

**DOI:** 10.3389/fpsyg.2022.998321

**Published:** 2022-11-17

**Authors:** Samantha R. O’Connell, Jessica E. Nave-Blodgett, Grace E. Wilson, Erin E. Hannon, Joel S. Snyder

**Affiliations:** ^1^Caruso Department of Otolaryngology, Head and Neck Surgery, Keck School of Medicine of USC, University of Southern California, Los Angeles, CA, United States; ^2^Department of Psychology, University of Nevada, Las Vegas, NV, United States

**Keywords:** auditory perception, musical sophistication, dance sophistication, groove, online studies

## Abstract

Listening to groovy music is an enjoyable experience and a common human behavior in some cultures. Specifically, many listeners agree that songs they find to be more familiar and pleasurable are more likely to induce the experience of musical groove. While the pleasurable and dance-inducing effects of musical groove are omnipresent, we know less about how subjective feelings toward music, individual musical or dance experiences, or more objective musical perception abilities are correlated with the way we experience groove. Therefore, the present study aimed to evaluate how musical and dance sophistication relates to musical groove perception. One-hundred 24 participants completed an online study during which they rated 20 songs, considered high- or low-groove, and completed the Goldsmiths Musical Sophistication Index, the Goldsmiths Dance Sophistication Index, the Beat and Meter Sensitivity Task, and a modified short version of the Profile for Music Perception Skills. Our results reveal that measures of perceptual abilities, musical training, and social dancing predicted the difference in groove rating between high- and low-groove music. Overall, these findings support the notion that listeners’ individual experiences and predispositions may shape their perception of musical groove, although other causal directions are also possible. This research helps elucidate the correlates and possible causes of musical groove perception in a wide range of listeners.

## Introduction

Moving to music is a common and pleasurable human behavior. Certain songs *groove* in that they encourage spontaneous movement and feelings of enjoyment (Madison, 2006; Madison et al., 2011; Janata et al., 2012; Matthews et al., 2020). Musical groove is recognized as a characteristic of songs encompassing genres such as jazz, pop, rock, hip hop, R&B, soul, and funk, made popular by artists like Stevie Wonder, Michael Jackson, and James Brown (Danielsen, 2006). The origins of *groove* are thought to be rooted in West African rhythms (Pressing, 2002). Early songs with groove are often associated with *swing*, a type of jazz music composed of “swinging” rhythms in which the beat is unevenly subdivided to sound like a lilt (Butterfield, 2010b). As music evolved, groove became an umbrella term describing a phenomenon in which musical rhythms invoke movement (Iyer, 2002). Songs with musical groove have become popular as naturalistic stimuli to study interactions between auditory and motor brain regions (Zatorre et al., 2007; Patel and Iversen, 2014). Listening to songs with groove can enhance performance on a range of physical tasks (Karageorghis and Terry, 1997; Styns et al., 2007; Buhmann et al., 2016) by eliciting longer strides and faster steps while walking (Leow et al., 2014), running (Edworthy and Waring, 2006), and rowing (Rendi et al., 2016). Even without accompanying movement, just listening to music with groove may have the power to excite neurons in the motor system (Wilson and Davey, 2002; Stupacher et al., 2013; Ross et al., 2016; Matthews et al., 2020; Martín-Fernández et al., 2021). As a result, musical groove listening is gaining traction as an enjoyable and therapeutic gait treatment for movement-related disorders such as Parkinson’s disease (Nombela et al., 2013; Leow et al., 2014).

To understand this musical phenomenon, researchers have studied the specific auditory components that may contribute to the sensation of groove (Stupacher et al., 2016a). Converging empirical evidence indicates that timing-based auditory properties such as a salient, low-pitched beat (Drake et al., 2000; Madison et al., 2011; Burger et al., 2012; Janata et al., 2012; Stupacher et al., 2016a; Hove et al., 2019), moderate rhythmic complexity (Temperley, 1999; Danielsen et al., 2014; Madison and Sioros, 2014; Sioros et al., 2014; Witek et al., 2014; Wesolowski and Hofmann, 2016; Witek, 2017; Matthews et al., 2019), and a medium tempo of about 120 beats per minute (MacDougall and Moore, 2005; Styns et al., 2007; Kornysheva et al., 2010; Janata et al., 2012; Leow et al., 2014; Michaelis et al., 2014; Stupacher et al., 2016a; Etani et al., 2018; Liu et al., 2018) have all been described as defining characteristics of musical groove. Beat-based musical elements may also activate neural motor networks. Listening to beat-based rhythms related to groove, without accompanying physical movement, engages auditory (Snyder and Large, 2005; Fujioka et al., 2009), prefrontal (Fukuie et al., 2022), and sensorimotor brain regions (Grahn and Brett, 2007; Grahn and Rowe, 2009, 2013; Fujioka et al., 2012). Additionally, listening to beats and rhythms can encourage kinesthetic movement by providing a temporal anchor to synchronize our bodies to the music (Iyer, 2002; Leman, 2012; Leow et al., 2021) and with one another (Kokal et al., 2011; Cirelli et al., 2014; Stupacher et al., 2017a,b). Performing synchronized movements can lead to arousal (Bowling et al., 2019), activation of reward networks (Menon and Levitin, 2005; Kokal et al., 2011; Zatorre, 2015; Matthews et al., 2020), and the release of feel-good neurotransmitters such as endorphins and oxytocin (Tarr et al., 2014, 2015; Josef et al., 2019), likely contributing to the overall enjoyable experience of being “in the groove” (Madison, 2006; De Bruyn et al., 2009; Janata et al., 2012).

There is a consensus that those with formal music training may have enhanced auditory perception (Kraus and Chandrasekaran, 2010; Strait et al., 2012, 2014, 2015; Kraus et al., 2014; Slater et al., 2015; Habibi et al., 2016) and emotional responses to music (Blood and Zatorre, 2001; Liu et al., 2018); however, there is a lack of consensus regarding how musical expertise may shape perception of musical groove. On one hand, research indicates that musicians’ perception of groove may be enhanced compared to non-musicians (Stupacher et al., 2013; Ross et al., 2016; Matthews et al., 2019). Musicians’ responsiveness to musical groove may be attributed to their ability to hear minute changes in acoustic elements better than non-musicians (Stupacher et al., 2016b). Musicians, compared to non-musicians, potentially have more awareness of musical elements important to musical groove such as harmonic complexity (Matthews et al., 2019), rhythmic complexity (Grahn and Rowe, 2009; Stupacher et al., 2017c; Matthews et al., 2019), tempo (Etani et al., 2018), syncopation (Madison and Sioros, 2014; Witek et al., 2014; Senn et al., 2018; Matthews et al., 2019), micro-timing deviations (Davies et al., 2013; Kilchenmann and Senn, 2015; Senn et al., 2016), and beat perception (Grahn and Rowe, 2009; Stupacher et al., 2017c; Nguyen et al., 2022). Additionally, relative to non-musicians, musicians’ motor systems may react more robustly to music with groove (Stupacher et al., 2013), possibly allowing for better balance control (Ross et al., 2016). This could arise from extensive training involving the synchronization of movements to the beat when producing musical sounds (Stupacher et al., 2013), resulting in stronger integration between perceptual and motor brain networks (Zatorre et al., 2007; Luo et al., 2012; Patel and Iversen, 2014; Martín-Fernández et al., 2021).

On the other hand, movement to music with groove may be a phenomenon experienced by a wide range of listeners (Madison, 2006; Madison et al., 2011; Janata et al., 2012), and thus formal expertise may be unnecessary for musical groove perception. For example, multiple studies have found no differences between musicians and non-musicians in their susceptibility to groove (Butterfield, 2010a; Frühauf et al., 2013; Hofmann et al., 2017). Most recently, Senn et al. (2019b) showed only marginal main effects of musical expertise on groove ratings when comparing musicians, amateur musicians, and non-musicians. In another study, non-musicians perceived music as groovier than musicians (Witek et al., 2014). Across these studies, musicians and non-musicians tend to agree on which songs are more or less “groovy”; however, their musical experiences may drive their preference for groove genres with higher or lower levels of musical complexity. For example, while musicians may rate more complex music, like jazz and funk, to be “groovier” (Pressing, 2002; Matthews et al., 2019), non-musicians may be inclined to rate pop and rock higher in groove because it is less complex and more familiar (Senn et al., 2021a). Taken together, factors such as innate biological traits, musical preferences, and musical exposure, rather than musical skills gained from playing an instrument, may have equal or greater effects on how we perceive the groove.

While previous research has revealed brain and behavior enhancements due to music training (Kraus and Chandrasekaran, 2010; Skoe and Kraus, 2010; Strait and Kraus, 2011; Slater and Kraus, 2016), musicality varies within populations of those with and without musical expertise (Zatorre, 2013; Nave-Blodgett et al., 2021a,b). This may be because biological and environmental benefits may contribute to heightened musicality in both musicians and non-musicians. In some instances, musicality may be cultivated due to an availability of resources (Corrigall et al., 2013). In other instances, one’s musicality may be a predisposed trait (Peretz et al., 2007; Tan et al., 2014; Mankel and Bidelman, 2018) that remains somewhat hidden due to a lack of financial or familial support (Schellenberg, 2015) or a lack of interest in learning to play music; however, some of these untrained individuals may become avid music appreciators and develop similar skills to musicians through hours of listening or other activities such as playing music video games (Pasinski et al., 2016). Furthermore, in both musicians and non-musicians, musical ability (Swaminathan and Schellenberg, 2018) and appreciation for certain types of music may be dictated by one’s personality (McCrae, 2007; Luck et al., 2010; Nusbaum et al., 2014; Colver and El-Alayli, 2015; Swaminathan and Schellenberg, 2018; Kuckelkorn et al., 2021) and music preferences (Madison, 2006; Salimpoor et al., 2013; Wesolowski and Hofmann, 2016; Madison and Schiölde, 2017; Senn et al., 2019b, 2021a; Kowalewski et al., 2020). Therefore, there is a growing need to understand *individual differences* in music perception that are not based on formal music training.

Groove has often been studied in the context of *music* performance: playing the music of a particular genre (e.g., jazz and funk), how the music is performed, or the enjoyable sensation of being “in the pocket” when musicians synchronize with the music and with one another (Berliner, 1994; Hosken, 2021). Historically, however, much of music was written for the purposes of *dancing* to music. For instance, songs from music genres known for groove rhythms, such as jazz or Afro-Cuban music, were first composed to accompany dance forms such as tap dance, swing dance (Madison, 2006), and Latin dances (Hughes, 2001). Oftentimes, it is hard to explain the feeling of groove without mentioning “movement” or “dancing.” While there is an undeniable connection between musical groove and dance (Merker, 2014; Fitch, 2016), there is a surprising dearth of empirical studies investigating the influence of dance experience on musical groove perception (Bernardi et al., 2017), or even general music perception.

As is the case with musical listening skills, dance-related skills may be hard to predict. Some dancers, like ballerinas, may possess years of professional training with a dance company while others may have years of self-taught experience dancing socially in a club. With the rise of social media, dance access has also become more widespread. Today, anyone with access to phone applications like TikTok can create, share, and learn dance choreography without having any prior experience. Dance experience or expertise may also be hard to assess because it can be difficult to disassociate from musical experience. For instance, tap dance straddles the fine line of being both music and dance because the art form equally values the importance of rhythmic sounds and movement. For this reason, many tap dancers identify as both musicians and dancers (Hill, 2010). In fact, the division of music and dance seems be a Western-focused mindset (Trehub et al., 2015). For example, in Nigeria and in India the very same term (*nkwa* and *sangeet*, respectively) is used for musical performance and dance (Balkwill and Thompson, 1999; Clayton, 2000). As the term *groove* itself is at the intersection of dance and music, it is important to study the influence of dance experience on musical groove perception regardless of one’s dance experience or how dance is identified.

Trained dancers, compared to non-dancers or non-trained dancers, may possess heightened functioning of sensorineural networks that may enhance their perception of musical groove. For instance, those with dance training show increased cortical thickness in superior temporal brain regions compared to non-experts (Karpati et al., 2017): these regions are vital to the auditory-motor integration network used during music listening and production (Bangert et al., 2006; Zatorre et al., 2007; Gordon et al., 2018). Additionally, trained dancers reveal enhancements in sensorimotor integration (Karpati et al., 2016) and appear to outperform non-trained dancers and non-musicians in audiovisual beat perception and production tasks (Nguyen et al., 2022). Furthermore, trained dancers, like trained musicians, show cortical phase synchrony in beta and gamma frequency bands during passive viewing of dance with music (Poikonen et al., 2018). These frequency bands have been implicated in musical beat encoding and auditory-motor brain interactions (Fujioka et al., 2009). Together, these studies suggest that dancers may exhibit training-induced neuroplasticity in sensorimotor regions that may engender heightened perception of the musical beat- a crucial component of musical groove.

Although dance expertise may hone music perception, feeling the groove may not be dependent on having superior perceptual or motor skills. Instead, the pleasure we feel from listening to music with groove may depend on our physical movement with music. For instance, those without formal dance training felt the most pleasure and arousal when moving spontaneously to high-groove music compared to low-groove music or when listening to music without movement (Bernardi et al., 2017). This may be because moving to music helps us understand the beat and meter through embodiment (Phillips-Silver and Trainor, 2006, 2008; Leman, 2012; Chemin et al., 2014; Lee et al., 2015). The habit of moving to music may also facilitate enjoyment of music with groove. Head movements to the beat of the music produce vestibular self-stimulated responses that may play an integral role in the understanding of musical beat (Phillips-Silver and Trainor, 2007; Todd and Lee, 2015), and meter (Phillips-Silver and Trainor, 2008; Trainor et al., 2009), and may activate brain circuits involved in reward (Todd and Lee, 2015; Reybrouck et al., 2019).

Additionally, high-groove music can strengthen the link between beat and movement because it tends to be syncopated (Janata et al., 2012; Witek, 2017; Witek et al., 2017), and the experience of syncopation depends on a strong, internally maintained beat (Pressing, 2002; Keller and Schubert, 2011; Sioros et al., 2014; Witek and Clarke, 2014). Knowing the locations of beats in time can help us synchronize our movements with the music and with others (De Bruyn et al., 2009). Past experiences moving to the music may also facilitate meter awareness. Those without formal dance training, but with experience dancing specific choreography, were better at tapping along to the music’s beat than those who did not learn the choreography (Lee et al., 2015). Furthermore, dance familiarity can be acquired through observation. Frequent spectators of dance, compared to novice dance spectators, showed increased corticospinal excitability as they viewed the form of dance with which they were most familiar (Jola et al., 2012). What is unclear, however, is whether these increases in meter perception and motor activation due to repeated dance observation translate to a heightened perception of musical groove. Therefore, there is a great need for investigations that directly study differences in music perception in those with varying degrees of dance experience.

In the present study, we investigated how musical and dance sophistication may influence musical groove perception in adult listeners with a wide range of artistic experiences. The first aim of this investigation was to understand how variations in *musical sophistication* predict musical groove perception. Specifically, we measured how both objective and subjective (self-reported) components of musical sophistication predict musical groove ratings. Musical sophistication is the possession of heightened music skills and engagement, and contains attributes such as musical understanding, appreciation, evaluation, and communication alongside skills such as playing an instrument, improvisation, and possessing a sense of rhythm and pitch (Hallam and Prince, 2003; Hallam, 2010; Müllensiefen et al., 2014). Objective components were perceptual musical skills measured using The Profile for Music Perception Skills (Law and Zentner, 2012; Zentner and Strauss, 2017) and the Beat and Meter Sensitivity Task (Nave-Blodgett et al., 2021a,b). Subjective components were measured using the Goldsmiths Musical Sophistication Index (Müllensiefen et al., 2014). We predicted that musical training, beat sensitivity, and measure sensitivity would be the most reliable predictors of musical groove perception, though other possible predictors could include active engagement, accent perception, or rhythm perception. It is vital to understand these subtleties in musicality across a wide range of listeners because musical groove’s likeability and effects on movement seem omnipresent (Madison, 2006; Madison et al., 2011; Janata et al., 2012), and thus potentially independent of skills that are only honed *via* formal music training (Leow et al., 2014).

The second aim of this study was to investigate the impact of *dance sophistication* on musical groove perception. Dance sophistication is the possession of heightened dance enjoyment, knowledge, or skills without necessarily undergoing formal dance training (Rose et al., 2020). We analyzed responses from the Goldsmiths Dance Sophistication Index (Rose et al., 2020), a new dance self-report assessment that distinguishes experience in dance participation from experience in dance observation to measure one’s overall dance comprehension. The present study marks one of the first investigations studying dance experience and musical groove perception. While there is little published work on how dance appreciation or experience may shape the way we perceive music with groove, we hypothesized dance training to be a strong predictor of musical groove perception in this model. Because we investigated listeners with varying degrees of dance experience, perception of musical groove in individuals with less dance experience may be more dependent on personal traits that make them more open to dancing in social settings.

## Materials and methods

### Participants

One hundred seventy-one adults completed the study. *A priori* power analyses using G^*^Power (Faul et al., 2009) determined that for a multiple regression model with seven predictors, data from 153 participants was effective in achieving a power (1–*β*) of 0.95 to detect a medium effect size (*f*^2^ = 0.15) at a statistical significance level of *α* = 0.05. Most participants were UNLV undergraduates enrolled in a psychology course (*n* = 146). The remaining participants were recruited by word of mouth, email, or by announcements posted on social media platforms (e.g., Facebook, Instagram, and Twitter). Twenty-three participants were excluded due to poor performance on the initial headphone check (see “Headphone check” for details); eight participants were excluded due to incorrect answers on compliance checks (see “Compliance check” for details); one participant was excluded due to an excessively noisy environment while completing the study; and 15 participants were excluded due to issues loading the stimuli. The final 124 participants were between the ages of 18–44 years old (*M* = 22.6 years, *SD* = 5.77 years, females = 80) and had no history of learning, neurological, or motor disorders. Power analyses using G*Power (Faul et al., 2009) determined this sample size was effective in achieving a power (1–*β*) of 0.885 to detect a medium effect size (*f*^2^ = 0.15) at a statistical significance level of *α* = 0.05. While musicians and dancers were not actively recruited for the present study, participants reported varying degrees of music and dance experience (see Table 1 for detailed music and dance experience).

**Table 1:** Participants’ musical and dance experience: self-reported category of expertise.

*n* (%)		Musical experience					
		NE	OM	RM	SAM	PM	Total
Dance experience	NE	37 (29.8%)	31 (25%)	7 (5.6%)	3 (2.4%)	2 (1.6%)	80 (64.5%)
	OD	12 (9.6%)	7 (5.6%)	3 (2.4%)	2 (1.6%)	0 (0%)	8 (6.4%)
	RD	2 (1.6%)	5 (4%)	1 (0.8%)	0 (0%)	0 (0%)	8 (6.4%)
	SAD	4 (3.2%)	3 (2.4%)	2 (1.6%)	1 (0.8%)	0 (0%)	10 (8.1%)
	PD	0 (0%)	1 (0.8%)	0 (0%)	1 (0.8%)	0 (0%)	2 (1.6%)
	Total	55 (44%)	47 (37.9%)	13 (10.4%)	7 (5.6%)	2 (1.6%)	124 (100%)

No experience (NE) = have no experience playing/participating. Occasional musician (OM)/Occasional dancer (OD) = less than weekly practice/participation. Recreational musician (RM)/Recreational dancer (RD) = weekly practice or recreational playing/performance. Serious Amateur Musician (SAM)/Serious Amateur Dancer (SAD) = extensive commitment to practice and/or recreational music or dance activity. Professional musician (PM)/Professional dancer (PM) = paid to perform and/or teach music or dance. Values are expressed as *n* (% of reported sample).

### Procedure

All testing was implemented online using Qualtrics (Qualtrics, Provo, UT, United States) and LimeSurvey (LimeSurvey, Hamburg, Germany). Participants followed an internet link to access the experiment (link can be found on the project’s Open Science Foundation Repository).^1^ Participants were required to sign a consent form before beginning the study. Participants were asked to complete the experiment on a computer over headphones in a quiet environment. Participants proceeded through the study beginning with the most difficult and attentionally taxing measures, described below in order of administration. Participants were offered opportunities to take short breaks after each test and subtest. Total test time was 60–90 min.

#### Headphone check

To ensure that participants were using headphones as requested, and could hear the auditory stimuli clearly, they completed a short assessment prior to beginning the experiment. In each trial, participants were presented with three tones and asked to indicate which was the quietest: the correct answer could only be discerned if the individual was wearing headphones rather than listening in free-field (Woods et al., 2017). We excluded data from participants who did not correctly answer at least five out of the six trials.

#### Profile for music perception skills

First, participants completed the short version of the Profile for Music Perception Skills (PROMS; Zentner and Strauss, 2017). This music aptitude battery objectively measures perceptual musical skills across multiple modalities in both musically trained and untrained individuals (Law and Zentner, 2012). We administered the Rhythm, Embedded Rhythm (rhythm-to-melody), Tempo, and Accent subtests because of their theorized importance to the feeling of musical groove (Witek et al., 2014; Etani et al., 2018; Matthews et al., 2019; Fukuie et al., 2022) and their robustness against noisy testing environments (Zentner and Strauss, 2017). We also chose to use the Melody subtest as an exploratory measure as previous research has yet to report that melody influences musical groove. Each subtest consists of eight to ten trials with a total testing time of 25 min. In each subtest, a trial consisted of a standard auditory stimulus (played twice) followed by one comparison auditory stimulus. Participants indicated (1) if the comparison stimulus was the same as the standard stimulus and (2) how confident they were in their answer.

The Melody subtest assessed the ability to recognize either tonal (easy) or atonal (difficult) melodies. Two-bar, eighth-note melodies were played by a MIDI harpsicord monophonically in 4/4 time. In different trials, one note of the comparison melody would differ from the reference melody by one semitone. The Rhythm subtest assessed the ability to recognize percussive rhythmic motifs. Two-bar phrases played equally accented in 4/4 time were composed of quarter, eighth, and sixteenth notes. Trials varied in difficulty by where the rhythmic deviant was located between the reference and comparison stimuli (i.e., easy trials had rhythmic deviants presented on downbeats and hard trials had rhythmic deviants presented on up-beats). The Embedded Rhythm subtest assesses the ability to recognize a percussive rhythmic motif when it is presented as part of a melody. Two-bar monophonically played and equally accented phrases in 4/4 time were composed of eighth and quarter notes. The reference stimulus was presented as a simple rhythm while the comparison stimulus was presented as a tonal melody. Participants were asked to identify whether the rhythm of the melody in the comparison stimulus matched the rhythm of the reference stimulus. The Tempo subtest assessed the ability to discriminate the speed at which music is played. Reference and comparison stimuli were polyphonically played in 4/4 time. The comparison stimuli ranged in difficulty by being 1 BPM (difficult) to 7 BPM (easy) different from the reference stimulus. The Accent subtest assessed the ability to discriminate the relative emphasis given to certain notes in a rhythmic pattern. Across two identical rhythmic motifs presented in 4/4 time monophonically by a MIDI drum sound, accented notes were presented as 3 dB louder than non-accented notes. Easy trials had more accent variations between reference and comparison stimuli compared to moderate and difficult trials. More detailed information on these subtests can be found in Law and Zentner (2012).

#### Beat and meter sensitivity task

In the next task, participants completed the Short BMS, a brief version of the Nave-Blodgett et al. (2021a,b) Beat and Meter Sensitivity Task (BMS), presented *via* Qualtrics. The BMS uses naturalistic music stimuli to assess auditory beat and meter sensitivity in individuals with varying levels of musical expertise, and does not require familiarity with musical terms, theory, or notation. In the Short BMS, participants listened to brief excerpts of commercially-recorded ballroom dance music overlaid with a custom click track that either matched or mismatched the music at the beat and measure levels (four possible alignment conditions). The musical excerpts were taken from six ballroom dance pieces, three of which were scored in 3/4 time (triple meter) and three of which were scored in 4/4 time (duple meter). The click track could fully match the beat and measure of the musical excerpt (beat matching/measure matching; e.g., a click track in 4/4 paired with a musical excerpt in 4/4), match the beat but not the measure (beat matching/measure mismatching; e.g., a click track in 3/4 paired with a musical excerpt in 4/4 where the beat of the click track and music aligns), match the measure of the music but not the beat (beat mismatching/measure matching; e.g., a click track in 3/4 paired with a musical excerpt in 4/4 where the measure-level downbeat matches but the beat does not), or not match either the beat or measure of the music (beat mismatching/measure mismatching; e.g., a click-track in 3/4 with a beat-level tempo 15% faster or slower than the musical excerpt in 4/4). Please consult the Open Science Foundation Repository^2^ for methods and stimulus information specific to the Short BMS, and Nave-Blodgett et al. (2021a,b) for general methods.

After listening to each musical excerpt and click track pairing, participants rated the fit of the click track to the music using a four-point Likert-type scale ranging from 1 (“Not Well at All”) to 4 (“Very Well”). Participants were given four practice trials to experience the stimuli and the rating scale prior to starting the experimental portion of the Short BMS. The Short BMS consists of 30 pairs of musical excerpts/click tracks. The musical excerpt/click track pairings were three musical measures long, which translated to approximately 6–8 s per trial. The task took approximately 10 min for participants to complete. The Short BMS results in two scores per participant, a *beat sensitivity score* that indicates participants’ ability to distinguish between beat-matching and beat-mismatching metronomes, and a *meter sensitivity score* that indicates participants’ ability to distinguish between metronomes that fully match the beat and measure of the music and those metronomes that match the beat of the music but not the measure.

#### Musical groove judgment task

Following the Short BMS, participants completed the Musical Groove Judgment Task (MGJT). Participants listened to 15-s clips of 10 high-groove (HG) and 10 low-groove (LG) songs and made judgments on what they heard. The ten songs rated highest in groove and the ten songs rated lowest in groove were chosen for this study from the Janata et al. (2012) music library (see Table 2 for complete song list). In this task, *groovy* was defined as how much a song makes you want to dance. On a seven-point Likert scale, they answered the following questions: (1) “Is this song groovy? (i.e., does it make you want to dance?),” (2) “Did you enjoy this song?,” and (3) “Are you familiar with this song?.” Likert scale choices ranged from *Not groovy at all, I do not like it at all,* and *This song is not familiar at all* to *Very groovy, I like it very much,* and *This song is very familiar*, respectively. Stimuli were truncated to 15-s segments using Audacity 2.1.2 (Audacity Team, 2021) and normalized to be the same volume. As in Janata et al. (2012), song stimuli were segmented starting at ~ 45 s into the song. This task took about 5 min to complete (Table 3).

**Table 2:** Participants’ musical and dance experience: self-reported characteristics.

Characteristic	*n*	*M*	*SD*	Range
Age started music lessons	37	9.7	3.7	4–16
Years of music lessons	37	6.5	4.2	1–20
Age started music ensemble	50	11.7	2.2	5–16
Years of music ensemble	50	5.8	4.7	1–30
Average hours of daily playing	37	2.7	2.5	0.5–11
Age started dance lessons	39	9.4	6.8	2–35
Years of dance lessons	39	8.6	7.6	0.5–27
Average hours of daily dancing	23	3.0	2.0	0.25–8
Hours of music listening per week	124	15.0	14.4	0–70

Years musical and dance training, age started musical and dance training, and hours daily playing only include participants with relevant experience. Hours of music listening per week include all participants. Those with both music and dance experience are not included in the separate totals for musical and dance experience.

**Table 3:** Songs used in the musical groove judgment task.

Song name	Artist	Groove	Genre	Groove rating
Superstition	Stevie Wonder	High	Soul	108.7
It’s a Wrap	FHI (Funky Hobo #1)	High	Soul	105.9
Flash Light	Parliament	High	Soul	105.1
Lady Marmalade	Patti LaBelle	High	Soul	102.5
Up for the Downstroke	The Clinton Administration	High	Soul	102.4
Mama Cita	Funk Squad	High	Soul	101.6
Music	Leela James	High	Soul	101.1
If I Ain’t Got You	Alicia Keys	High	Soul	98.7
Sing, Sing, Sing	Benny Goodman	High	Jazz	97.4
In the Mood	Glenn Miller	High	Jazz	96.9
Space Oddity	David Bowie	Low	Rock	38.7
Ray Dawn Balloon	Trey Anastasio	Low	Rock	38.5
Druid Fluid	Yo-Yo Ma, Mark O’Connor, and Edgar Meyer	Low	Folk	38.1
Flandyke Shore	The Albion Band	Low	Folk	36.5
Citi Na GCumman	William Coulter and Friends	Low	Folk	35.2
Dawn Star	Dean Magraw	Low	Folk	34.8
Fortuna	Kaki King	Low	Folk	32.6
Beauty of the Sea	The Gabe Dixon Band	Low	Rock	32.1
Sweet Thing	Alison Brown	Low	Folk	30.9
Hymn for Jaco	Adrian Legg	Low	Folk	29.3

Groove = groove category (i.e., low or high). Groove rating values are derived from Janata et al. (2012).

#### Goldsmiths musical sophistication index

Upon completion of the MGJT, participants completed the Goldsmiths Musical Sophistication Index Self-Report Inventory (Gold-MSI), a 39-item psychometric instrument used to quantify the amount of musical engagement, skill, and behavior of an individual (Müllensiefen et al., 2013). The questions on this assessment are grouped into five subscales: Active Engagement, Perceptual Abilities, Musical Training, Singing Abilities, and Emotions (see Müllensiefen et al., 2013, 2014 for each subscale’s detailed question information). The Active Engagement subscale comprised questions that described a range of active musical engagement behaviors (e.g., “I keep track of new music that I come across (e.g., new artists or recordings)” or “I do not spend much of my disposable income on music”). The Perceptual Abilities subscale comprised questions that each represented the self-assessment of cognitive musical ability and music listening skills (e.g., “I can tell when people sing or play out of tune”). The Musical Training subscale combined questions involving the extent of self-reported musical training and practice (e.g., “I engaged in regular daily practice of a musical instrument including voice for __ years”), and the degree of self-assessed musicianship (“I would not consider myself a musician”). The Singing Abilities subscale comprised questions that reflected upon different self-reported skills and activities related to singing (e.g., “I am not able to sing in harmony when somebody is singing a familiar tune”). The Emotions subscale comprised questions describing self-reported behaviors that happen frequency in response to an external music source. These questions were not assessing planned behaviors or those that could change based on increased musical experience (e.g., “I hardly ever hum or sing along to music”). All items, except those assessing Musical Training, are scored on a seven-point Likert scale with choices that range from *Completely disagree* to *Completely agree*. The composite score of these five subscales makes up an individual’s General Musical Sophistication score (Müllensiefen et al., 2013). More details about the Gold-MSI can be found in Müllensiefen et al. (2013).

#### Goldsmiths dance sophistication index

After the Gold-MSI, participants completed the Goldsmiths Dance Sophistication Index (Gold-DSI), a 26-item standardized self-report instrument used to quantify individual differences in doing dance (i.e., participatory dance experience), watching dance (i.e., observational dance experience), and one’s knowledge about dance (Rose et al., 2020). Like the Gold-MSI, the Gold-DSI is designed to measure a wide range of dance skills, behaviors, and engagement in a general population (Rose et al., 2020). The Gold-DSI is comprised of two separate inventories: Participatory Dance Experience and Observational Dance Experience. The composite score of four subtests (Body Awareness, Social Dancing, Urge To Dance, and Dance Training) contribute to the Participatory Dance Experience score while the composite score on six separate questions comprises the Observational Dance Experience score (see Rose et al., 2020 for each subscale’s detailed question information). The questions were randomized per participant. The Body Awareness subscale consisted of items that ask about the degree of self-assessed movement and coordination (e.g., “I find it easy to learn new movements”). The Social Dancing subscale consisted of items describing self-reported behaviors about one’s time spent dancing with others and the emotions felt around dancing in public places (e.g., “If someone asks me to dance, I usually say yes”). The Urge To Dance subscale consisted of items describing self-reported physical and emotional responses to music related to dance and how much time spent dancing (e.g., “When I dance, I feel better”). The Dance Training subscale consisted of questions describing the extent of one’s formal dance experience and their self-assessed level of dance ability (e.g., “I have taken regular dance classes at least once a week for __ years”). The Observational Dance Experience subscale consisted of items that ask the extent to which one self-reports watching dance in-person or on TV/online and the emotions felt when watching dance (e.g., “I like watching people dance”). All items, except those assessing Dance Training, are scored on a seven-point Likert scale with choices that range from *Completely disagree* to *Completely agree*. More details about the Gold-DSI can be found in Rose et al. (2020).

#### Demographics

The final task participants completed was a demographics questionnaire that asked questions about health history, music experience, dance experience, exercise, and engagement with music listening.

#### Compliance check

Throughout the study, we utilized a set of previously published questions to ensure participants were adequately attending to the experimental task (Mehr et al., 2018). Within each experimental block, participants were asked one time to answer the following question: “What color is the sky? Please answer this incorrectly, on purpose, by choosing red instead of blue.” The possible response options were “Green,” “Blue,” “Red,” or “Yellow”. The correct response was only presented in each answer slot once. This question was presented a total of five times. Participant who did not select “Red” to all five questions were excluded from analysis.

After the completion of the experiment, participants were asked to answer compliance questions to ensure that the experiment was completed with effort in an environment with minimal distraction. The first question stated, “People are working on this task in many different places. Please tell us about the place you were at when working on this task. Please answer honestly.” Response options were (1) “I worked on this study in a very noisy place,” (2) “I worked on this study in a somewhat noisy place,” (3) “I worked on this study in a somewhat quiet place,” or (4) “I worked on this study in a very quiet place”. Those who answered the question with “I worked on this study in a very noisy place” or “I worked on this study in a somewhat noisy place” were excluded from analysis. The second question asked, “Please tell us if you had difficulty loading the sounds. Please answer honestly.” From “Yes” or “No” response choices, any participant who responded with “Yes” was excluded from analysis. The final question asked, “How carefully did you complete this experiment? Please answer honestly.” From the response options of (1) “Not at all carefully,” (2) “Slightly carefully,” (3) “Moderately carefully,” (4) “Quite carefully,” and (5) “Very carefully,” those who answered with “Not at all carefully,” “Slightly carefully,” or “Moderately carefully” were excluded from analysis.

### Statistical design

All data for this study can be found here: https://osf.io/g3y7c/. The main analysis was a stepwise multiple linear regression that predicted *musical groove sensitivity score*, which is the difference between mean high-groove music ratings and mean low-groove music ratings (*M*_high-groove music_ – *M*_low-groove music_). Musical groove sensitivity scores can range from – 6 to + 6. Those with higher musical groove sensitivity scores (score = 4–6) perceive greater differences between high- and low-groove songs. Those with lower musical groove sensitivity scores (score = 1–3) either perceive less differences between high-and low-groove songs or no differences (score = 0) between the two groove types. A score evaluating the difference between high- and low-groove music was employed rather than separately regressing ratings on high- and low-groove songs for two reasons. First, a difference score uses all the groove rating data in a single outcome measure. Second, a difference score controls for response bias or how individual participants use the subjective rating scale for “grooviness.” For example, two participants might provide different numbers for the same subjective amount of groove for a low-groove song, but it is assumed that the increased rating they would each provide for a high-groove song would reflect an accurate measure of their sensitivity for the difference between low-groove and high-groove songs. Overall, this allows for a more nuanced measurement of musical groove that captures how individuals differently rate high- and low-groove music.

A bidirectional stepwise linear regression analysis was performed in the R statistical software environment (R Core Team, 2022) to assess how subtests of the Gold-MSI, the Gold-DSI, the PROMS, and the Short BMS may predict musical groove sensitivity. First, we used the *stepAIC( )* function from the MASS package to choose predictors for a best-fit model based on the Akaike Information Criterion (AIC): a measure of fit that estimates the quality of each model. This automatic function evaluates models in parallel to avoid overfitting of data and cherry-picking of predictors (Venables and Ripley, 2002). Then, we chose the best-fit model based on the lowest AIC value and ran a multiple linear regression analysis using the *lm( )* function on the resulting automatically chosen predictors.

Analysis of variance (ANOVA) evaluating musical groove, familiarity, and likeability ratings and Pearson’s *r* correlation coefficients between the predictor and criterion variables for the resulting stepwise multiple regression analysis were calculated in SPSS 28 (IBM, Chicago, IL, United States). The ANOVA for musical groove, likeability, and familiarity ratings was replicated from Janata et al. (2012) and was conducted with the musical excerpt as a case (i.e., data averaged across participants for each excerpt) rather than the participant. This was intentional to validate this dataset against the original findings of Janata et al. (2012).

## Results

### Relation of musical groove, likeability, and familiarity

**Figure 1:**
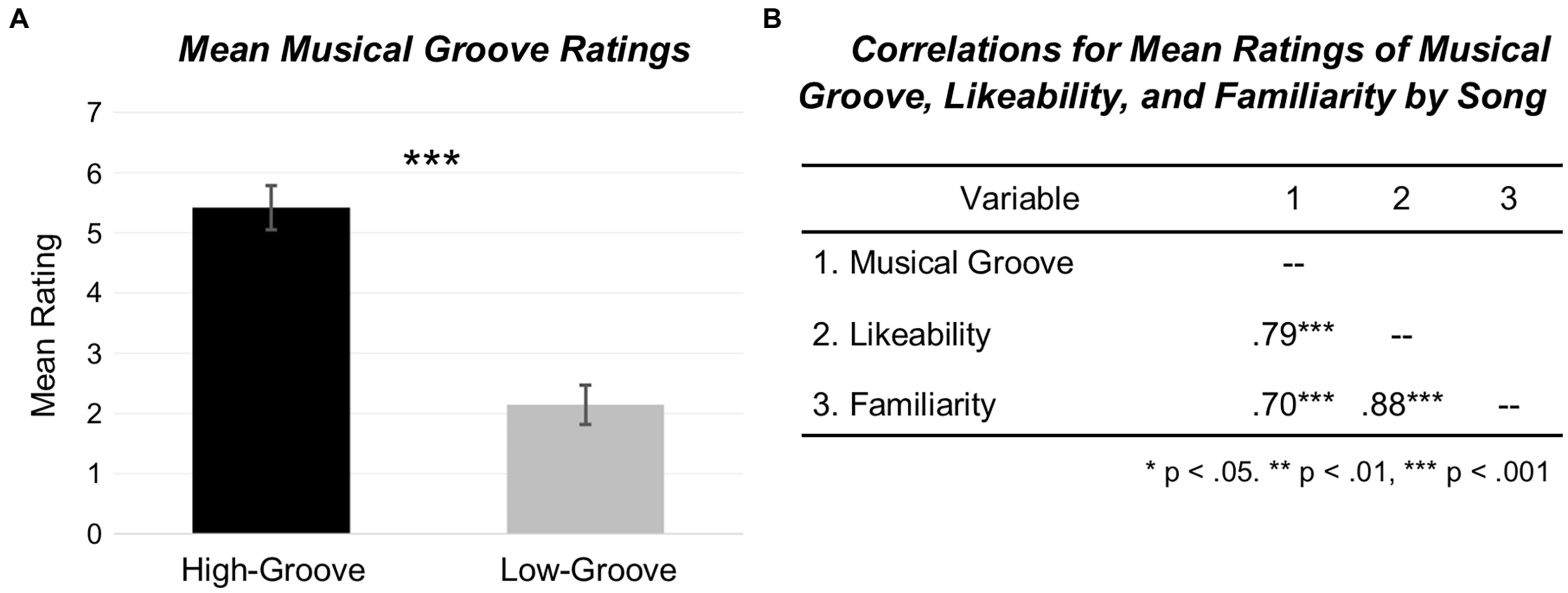
Mean musical groove ratings and correlations for musical groove, likeability, and familiarity. **(A)** Bar graphs of mean musical groove ratings (*N* = 20; high-groove = 10, low-groove = 10) based on musical excerpt as a case (i.e., data averaged across participants for each excerpt). Error bars indicate 95% confidence intervals. Results reveal statistically significant differences between high-groove (black) and low-groove (grey) mean song ratings *F*_1,18_ = 226.02, *p* < 0.001. **(B)** Relationships between mean musical groove ratings, mean likeability ratings, and mean familiarity ratings (*N* = 20; high-groove = 10, low-groove = 10). Results show statistically significant positive correlations between musical groove and likeability ratings, musical groove and familiarity ratings, and likeability and familiarity ratings.

Replicating prior work (Janata et al., 2012), a one-way analysis of variance (ANOVA) with a two-tailed alpha level of 0.05 was conducted with musical excerpt as a case (i.e., data averaged across participants for each excerpt). The results confirmed that listeners in the Musical Groove Judgment Task gave higher groove ratings to high-groove (*M* = 5.42, CI = 5.05, 5.79) than to low-groove excerpts (*M* = 2.14, CI = 1.81, 2.47), *F*_1,18_ = 226.02, *p* < 0.001, *η*^2^ = 0.926 (see Figure 1A). This confirms that the songs identified in this study, borrowed from Janata et al. (2012), were categorized correctly as high-groove or low-groove by the researchers and confirmed by listener ratings. There were also statistically significant positive correlations between mean musical groove ratings and likeability ratings, *r* (18) = 0.79, *p* < 0.001; groove and familiarity ratings, *r* (18) = 0.70, *p* < 0.001; and familiarity and likeability ratings, *r* (18) = 0.88, *p* < 0.001 (see Figure 1B).

### Stepwise multiple linear regression analysis

We first entered a total of 17 predictors into the stepwise linear regression model. The predictors were the total scores of each of the Gold-MSI subscales (i.e., Active Engagement, Perceptual Abilities, Emotions, Singing Abilities, and Musical Training), the total scores of each of the Gold-DSI subscales (i.e., Body Awareness, Social Dancing, Urge To Dance, and Dance Training, and Observational Dance Experience), the total scores of each PROMS subtest (i.e., Melody, Tempo, Accent, Rhythm, and Embedded Rhythm), and the total scores on each of the Short BMS measures (i.e., beat sensitivity and measure sensitivity). After running the *stepAIC( )* function, we chose the best fitting model based on the lowest AIC value (AIC = −22.612). The selected model for analysis contained seven predictors: three predictors from the Gold-MSI (Perceptual Abilities, Musical Training, and Emotions), two predictors from the Gold-DSI (Social Dancing and Dance Experience), one predictor from the PROMS (Accent), and one predictor from the Short BMS (beat sensitivity). We regressed these predictors using the *lm()* function to find that Perceptual Abilities, Musical Training, and Social Dancing scores significantly predicted musical groove sensitivity, *F* (7, 116) = 5.091, *p* < 0.001, *R*^2^ = 0.24, adj. *R*^2^ = 0.19 (see Table 4). Emotions, Dance Training, Accent and beat sensitivity scores were not statistically significant predictors of groove sensitivity, *ps* > 0.05. Multicollinearity was assessed: VIF were below 2.50 suggesting no presence of multicollinearity (Kutner et al., 2005). The accompanying correlations between predictor and criterion variables can be found in Table 5.

**Table 5:** Correlations between variables in stepwise multiple linear regression analysis.

Variable	1	2	3	4	5	6	7	8
1. Musical Groove Sensitivity Score	–							
2. Gold-MSI Perceptual Abilities	0.31^**^	--						
3. Gold-MSI Musical Training	0.02	0.55^***^	–					
4. Gold-MSI Emotions	0.31^**^	0.53^***^	0.33^***^	–				
5. Gold-DSI Social Dancing	0.21^*^	0.19^*^	0.05	0.15	–			
6. Gold-DSI Dance Training	0.05	0.37^***^	0.35^***^	0.27^**^	0.52^***^	–		
7. BMS Beat Sensitivity	0.29^**^	0.36^***^	0.28^**^	0.37^***^	0.16	0.22^*^	–	
8. PROMS Accent	0.20^*^	0.28^**^	0.37^***^	0.19^*^	−0.003	0.17	0.43^***^	–

* *p* < 0.05;

** *p* < 0.01;

*** *p* < 0.001.

**Table 4:** Stepwise multiple linear regression results.

Variable	*B*	97.5% CI for *β*		SE_B_	*β*	*t*	*p*
		LL	UL				
Gold-MSI Perceptual Abilities	0.03	0.24	0.29	0.01	0.27	2.46	**0.015** ^*^
Gold-MSI Musical Training	−0.02	−0.24	−0.20	0.01	−0.22	−2.15	**0.034** ^*^
Gold-MSI Emotions	0.03	0.14	0.21	0.02	0.17	1.78	0.077
Gold-DSI Social Dancing	0.02	0.18	0.23	0.01	0.21	2.17	**0.032** ^*^
Gold-DSI Dance Training	−0.03	−0.21	−0.15	0.02	−0.18	−1.73	0.087
BMS Beat Sensitivity	0.20	−0.15	0.42	0.15	0.13	1.38	0.171
PROMS Accent	0.09	0.03	0.26	0.06	0.14	1.51	0.133

Criterion variable, musical groove sensitivity score. AIC = −22.612, *F*(7, 116) = 5.091, *p* < 0.001, *R*^2^ = 0.24, adj. *R*^2^ = 0.19. *B*, unstandardized regression coefficients. CI, confidence interval. LL, lower limit. UL, upper limit. *β*, standardized regression coefficients. ^*^p < 0.05 and ^**^p < 0.01. Bolded values emphasize the significant predictors found in the stepwise regression model.

## Discussion

The present study investigated individual differences in music and dance characteristics that may contribute to musical groove perception. Specifically, this online experiment examined 17 potential predictors and assessed how facets of musical sophistication, dance sophistication, and performance on music-based perceptual tasks influenced individuals’ sensitivity to musical groove. Although previous studies focused on the acoustic components of music (Witek et al., 2014; Stupacher et al., 2016a; Senn et al., 2017, 2018) and the way music is performed that makes the music itself “groovy” (Hurley et al., 2014; Witek and Clarke, 2014; Kilchenmann and Senn, 2015; Senn et al., 2016), here we chose to ask how individual differences in listeners’ experiences, training, and perceptual skills might shape the way they experience musical groove. Our study is novel in that we use a new measure, the *musical groove sensitivity score*, which can be used in a regression framework to examine the relationship between an individual’s groove perception and other individual difference measures.

In general, our participants agreed on ratings of musical groove, familiarity, and likeability. Songs previously rated as high and low in musical groove by listeners in Janata et al. (2012) were rated similarly by the listeners in the present study. Specifically, our listeners rated high-groove music as being significantly more “groovy” than low-groove music. As in Janata et al. (2012), our participants also rated high-groove songs as more familiar and more likeable than low-groove songs. Musical groove ratings, familiarity ratings, and likeability ratings all had strong, positive relationships with one another.

Using an AIC-based stepwise model selection, seven out of 17 possible predictors from subtest scores of the Gold-MSI, Gold-DSI, PROMS, and Short BMS were chosen to predict the musical groove sensitivity score. The seven selected predictors (Gold-MSI Perceptual Abilities, Gold-MSI Musical Training, Gold-MSI Emotions, Gold-DSI Dance Training, Gold-DSI Social Dancing, PROMS Accent, and Short BMS beat sensitivity) together accounted for 24% of the variance in musical groove sensitivity score. Of these predictors, self-reported Perceptual Abilities, Musical Training, and Social Dancing scores separately predicted musical groove difference ratings compared to the other predictors in the model. Emotions, Dance Training, Accent, and beat sensitivity scores did not significantly predict the difference between high-and low-groove music ratings.

### Perceptual abilities and musical training

The Perceptual Abilities subtest of the Gold-MSI is comprised of self-reported views of song recognition, tonal perception, genre identification, and how well one can judge others’ musical abilities (Müllensiefen et al., 2014). Pearson *r* correlations reflected a positive relationship between Perceptual Abilities and musical groove sensitivity scores indicating that those who think they are good at judging others’ musical abilities, identifying musical genres, recognizing familiar music, and spotting mistakes in performances tend to rate high-and low-groove music more distinctly. Perceptual Abilities also had significant positive correlations with Gold-MSI Musical Training, Gold-MSI Emotions, Gold-DSI Dance Training, Gold-DSI Social Dancing, PROMS Accent, and Short BMS beat sensitivity.

The Musical Training subtest of the Gold-MSI is comprised of self-reported views of musicianship as well as quantitative measurements of practice time, formal training, and instrument type (Müllensiefen et al., 2014). Pearson *r* correlations reflected a barely positive relationship between Musical Training and musical groove sensitivity scores indicating those that consider themselves to be musicians, those that are complimented more often on performance quality, and those who report more hours of daily practice, greater years of formal music training, and increased numbers of instruments played rated high-and low-groove music more distinctly. Musical Training also had significant positive correlations with Gold-MSI Perceptual Abilities, Gold-MSI Emotions, Gold-DSI Dance Training, PROMS Accent, and Short BMS beat sensitivity, but was not significantly correlated with Gold-DSI Social Dancing.

In the stepwise regression model, the Perceptual Abilities score was a significant positive predictor of musical groove sensitivity score. Interestingly, Musical Training score was a significant *negative* predictor of musical groove sensitivity score. This regression result was surprising considering that when correlated on its own, Music Training score has a barely *positive* association with musical groove sensitivity score. It is only when other predictors are considered in the regression model, however, that Musical Training has a negative regression coefficient.

These unpredicted results may be connected to the positive association found between Gold-MSI Perceptual Abilities and Musical Training subtest scores. Individuals who possess more honed perceptual abilities may have more musical training. Five out of seven questions on the Gold-MSI Musical Training subtest ask about quantitative hours of practice and years of training. Therefore, it seems that music training quantity is highly weighted in the final subscale score and is designed to identify individuals with formal music training. Questions that comprise the Gold-MSI Perceptual Abilities subtest, such as judging musical abilities and spotting mistakes during performances, are also some of the many skills that are taught and honed when formally learning to sing or play an instrument at a high level.

Additionally, those with formal music training may rate songs with groove differently from those without formal training. Previous research has shown that musicians have rated more complex music, like jazz and funk, to be “groovier” (Pressing, 2002; Matthews et al., 2019) while non-musicians have rated less complex music, such as pop and rock, higher in groove (Senn et al., 2021a). This may be due to musicians understanding and appreciating more complex music and how familiar musicians are with their genre of expertise. For instance, a jazz musician may be better at differentiating between high- and low-groove music if they were making ratings across only jazz music as opposed to rating only pop music. What is missing from the Gold-MSI, however, is an assessment about the *type* of music these musicians play. While this specific examination did not collect sufficient demographic data about the type of genre musicians claimed expertise in, future research should consider comparing musical groove sensitivity scores across a variety of musicians with different types of expertise to see if familiarity with a certain genre can drive musical groove ratings.

Conversely, possessing greater perceptual abilities may be linked to more *experience* with music (e.g., avid listening or attending concerts), or associated with self-taught, informal training (e.g., funk players with 20+ years of band experience), but may not be indicative of *formal* music training (e.g., conservatory-trained classical musicians). Müllensiefen et al. (2014) found that both the Gold-MSI Perceptual Abilities and Music Training subscale scores had strong positive associations with perceptual musical skills tasks such as the Gold-MSI Beat Perception and Melody Memory tests. Our data also supports this notion with positive, significant relationships between the Gold-MSI Perceptual Abilities, Gold-MSI Musical Training, Short BMS beat sensitivity, and PROMS Accent—two perceptual tasks that measure performance on music-related skills designed to potentially identify musical ability not necessarily honed through training.

Considering the positive and negative associations between Gold-MSI Perceptual Abilities and Musical Training in both the correlation and regression analyses, respectively, the regression result seems to imply that among those who score higher on Perceptual Abilities, avid music appreciators and those with *informal* music training may make greater distinctions between high- and low-groove music compared those with *formal* music training. For instance, the questions that make up the Gold-MSI Perceptual Abilities subtest ask about genre identification and recognition of familiar and novel songs. While these abilities can be learned through formal music training, they can also be refined through frequent music listening or informal music training. The high-and low-groove songs that were selected for this study belonged to differing genres: high-groove songs were previously categorized as belonging to soul and jazz genres while low-groove songs were previously identified as belonging to rock and folk genres (Janata et al., 2012). Avid music appreciators who have experience listening to a wide variety of music, or informally trained musicians with ample experience playing in funk and soul bands, may be able to easily identify high-and low-groove music genres as “dance” or “non-dance” songs, respectively, based on genre, but not necessarily on how much they make them want to dance. Frequent music listeners are also potentially better than music novices at recognizing familiar and unfamiliar music. Because songs higher in groove in this study and others (Janata et al., 2012; Senn et al., 2021a) were also rated as more familiar, these individuals may rate high- and low-groove music more distinctly based on familiarity rather than how much it makes them want to dance. Future research should consider matching high- and low-groove songs on genre and familiarity to further disentangle groove from familiarity and its associations with pleasurable movement.

It is also possible that the discrepancy found between the Gold-MSI Musical Training score and the musical groove sensitivity score in the correlation and regression analyses may be affected by suppressor variables in the stepwise regression. Suppressed variables are sometimes identified by being highly positively correlated with other significant predictors within the regression model but are not significantly positively correlated with the criterion variable. These types of predictors may therefore have different relationships with a criterion variable when doing simple correlation versus multiple regression (Pandey and Elliott, 2010). The Gold-MSI Musical Training predictor seemed to fit this mold: it is significantly positively correlated with Gold-MSI Perceptual Abilities but not with musical groove sensitivity and has a significant negative regression coefficient in the regression model. While this study was more exploratory in identifying potential variables that may predict musical groove sensitivity score, it is possible that removing the Musical Training score from the model may increase the magnitude in the relationship between other significant predictors and the criterion variable (Mackinnon et al., 2000).

### Social dancing

Social Dancing is a Gold-DSI subtest comprised of self-reported views of social dance enjoyment and engagement (Müllensiefen et al., 2014). Pearson *r* correlations reflect a positive relationship between Social Dancing and musical groove sensitivity scores, indicating those who have more engagement in social dancing, greater experience dancing with others, and heightened enjoyment participating in social dance rated high- and low-groove music more distinctly. Social Dancing also had significant positive correlations with Gold-MSI Perceptual Abilities and Gold-DSI Dance Training but was not significantly correlated with Gold-MSI Musical Training, Gold-MSI Emotions, PROMS Accent, and Short BMS beat sensitivity.

The stepwise regression model revealed the Gold-DSI Social Dancing score as a significant positive predictor of musical groove sensitivity score. Unexpectedly, Dance Training score was not a significant predictor of musical groove sensitivity score and when correlated on its own, Dance Training did not have a significant association with musical groove sensitivity score.

The Gold-DSI Social Dancing score may be a significant predictor of musical groove sensitivity scores because it assesses dance in the context of socialization and enjoyment: all previously reported descriptors of how people feel and act when hearing songs with groove (Janata et al., 2012). Fitch (2016) argues that “…core aspects of musical rhythm, especially ‘groove’ and syncopation, can only be fully understood in the context of their origins in the participatory social experience of dance” (p. 1). Considering the positive association between Gold-DSI Social Dancing and Dance Training scores, those who scored higher on Social Dancing may be individuals with extensive dance training. Oftentimes, classical dance forms such as ballet, modern, or lyrical are choreographed and performed to low-groove songs while social, contemporary, and percussive dance forms like jazz, tap, and hip-hop are performed to high-groove songs. Therefore, these individuals would be well-trained in evaluating what is considered “groovy” music based on the dance form with which the music is associated. It is also possible that those with more formal dance training also are more likely to go social dancing compared to those with less formal training. The Gold-DSI Dance Training subtest does gather quantitative information about formal dance training (e.g., years of involvement in formal dance classes); however, the Social Dancing subtest does not assess quantitative social dance experience (e.g., how many hours per week spent social dancing at a party or club), but rather the qualitative experience of dancing (e.g., “Dancing with other people is a great night out as far as I’m concerned”). While the Gold-DSI does not gather this information, future studies should investigate whether those with more formal dance training also spend more time social dancing.

Taking together the correlation and regression analyses between Dance Training, Social Dancing, and musical groove sensitivity, however, this regression analysis seems to indicate that among frequent social dancers, those with *less* formal dance training (e.g., those who attend clubs and parties to dance with friends) tend to hear greater differences between high- and low-groove music than those with more formal dance training (e.g., professional classical ballerinas). This seems to contradict our original prediction that Dance Training would be a significant predictor of musical groove sensitivity score. Those who scored higher on Social Dancing may not be formal dancers, but experienced non-trained dancers or dance appreciators who enjoy dancing with others as a form of bonding and socialization. Because songs with groove are often danced to in social settings, those who feel more comfortable dancing socially may have more familiarity with musical groove and as a result, are better at identifying differences between high- and low-groove music. Those who enjoy social dancing may also be people who have greater openness to experience or are more extraverted. Previous research has found that those who report more openness to experience also have more episodes of pleasurable esthetic chills to music (Colver and El-Alayli, 2015), which may suggest greater emotional connection to music. Those who self-report as being more extraverted also have greater local and global body movements, faster head speeds, and greater hand flux and hand distance when moving to music belonging to high-groove genres such as rock, jazz, Latin, techno, funk, and pop (Luck et al., 2010). This may indicate that those who enjoy dancing to music from high-groove genres may also engage in more movement while dancing, and as a result, have a more embodied representation of the music itself. Through movement, these individuals may develop a better sense of the beat and facilitate more enjoyment of groove through head movements that stimulate the vestibular system and reward networks (Phillips-Silver and Trainor, 2007, 2008; Reybrouck et al., 2019).

### Limitations and future directions

A limitation to the current study was the online format, which was chosen due to social-distancing restrictions during the COVID-19 pandemic which made in-person testing not feasible. For this reason we used subjective groove ratings, which may depend on individual participants’ interpretation of the word “groovy.” Although we defined groove for participants as “does it make you want to dance?,” it is nevertheless possible that to some extent their ratings reflect their associations between certain musical genres and the word “groovy.” Similarly, our measures of sensitivity to musical beat were based on subjective ratings of fit between a metronome and music. Collecting accurate temporal information or finger tapping data in online tasks is unreliable due to potential timing lags and lack of necessary equipment in everyday households. A future extension of this work could incorporate production tasks, such as a beat synchronization test in which participants tap along to music. It is possible that the ability to produce a beat accurately in time to music may be a more reliable predictor of hearing differences between high-and low-groove music than purely perceptual beat sensitivity.

This study explored sensitivity ratings of 10 high- and 10 low-groove songs. We selected a subset of songs that were exemplars of high and low-groove music based on previous work (Janata et al., 2012; Stupacher et al., 2013) while also considering the time needed to obtain good data in an online study without participant fatigue. This design did not allow us for time to include songs that have been previously rated as “mid-groove.” Future research should consider using a wider range of songs that capture high-, mid-, and low-groove music to obtain a more inclusive landscape of different musical genres and preferences to see how personal experiences and predilections can influence perceptions of songs with moderate groove.

## Conclusion

The present study investigated the influence of musical sophistication, dance sophistication, and musical perceptual abilities on musical groove perception. We found that perceptual abilities, musical training, and social dancing are significant predictors of rating differences between high-and low-groove music. Overall, our results indicate that the experience of groove may not be solely dependent on the way the music is written or performed but also shaped by listeners’ individual experiences and predispositions. Results from this investigation may help develop more objective assessments of dance skills that can measure dance ability in a wide array of individuals. Clinical implications of this research may help with the development of musical therapeutic tools for those diagnosed with movement impairments (e.g., Parkinson’s disease; Nombela et al., 2013; Krotinger and Loui, 2021) or developmental disorders (e.g., ADHD; Puyjarinet et al., 2017), who have a harder time moving to the beat compared to healthy and typically developing individuals, respectively.

## Data availability statement

The datasets presented in this study can be found in online repositories. The names of the repository/repositories and accession number(s) can be found at: https://osf.io/g3y7c/.

## Ethics statement

The studies involving human participants were reviewed and approved by the University of Nevada, Las Vegas Institutional Review Board. The participants provided their written informed consent to participate in this study.

## Author contributions

SO’C, EH, and JS: conceptualization. SO’C, JN-B, and JS: methodology. SO’C: formal analysis and data collection. SO’C and GW: investigation setup. SO’C and JN-B: writing—original draft preparation. SO’C, JN-B, EH, and JS: writing—review and editing. All authors contributed to the article and approved the submitted version.

## Funding

This research was supported in part by the University of Nevada, Las Vegas Foundation Board of Trustees Fellowship awarded to SO’C. The publication fees for this article were supported by the UNLV MSI Open Article Fund.

## Conflict of interest

The authors declare that the research was conducted in the absence of any commercial or financial relationships that could be construed as a potential conflict of interest.

## Publisher’s note

All claims expressed in this article are solely those of the authors and do not necessarily represent those of their affiliated organizations, or those of the publisher, the editors and the reviewers. Any product that may be evaluated in this article, or claim that may be made by its manufacturer, is not guaranteed or endorsed by the publisher.

## Footnotes

^1^ https://osf.io/g3y7c/

^2^ https://osf.io/8nfvq/
